# Cross-validation of two prognostic trauma scores in severely injured patients

**DOI:** 10.1007/s00068-020-01373-6

**Published:** 2020-04-22

**Authors:** Rolf Lefering, Stefan Huber-Wagner, Bertil Bouillon, Tom Lawrence, Fiona Lecky, Omar Bouamra

**Affiliations:** 1 Institute for Research in Operative Medicine (IFOM), Faculty of Health, University of Witten/Herdecke, Ostmerheimer Strasse 200 (Building 38), 51109 Cologne, Germany; 2 Department of Trauma and Orthopedic Surgery, Diakonie Hospital Schwaebisch Hall, Schwäbisch Hall, Germany; 3 Department of Trauma Surgery and Orthopedics, Cologne-Merheim Medical Center, Cologne, Germany; 4 Faculty of Biology, Medicine and Health, The Trauma Audit and Research Network, The University of Manchester, Salford, UK; 5 Centre for Urgent and Emergency Care Research (CURE), Health Services Research Section, School of Health and Related Research, University of Sheffield, Sheffield, UK

**Keywords:** Severe injuries, Trauma registry, Survival, Prognosis, Score, Outcome

## Abstract

**Introduction:**

Trauma scoring systems are important tools for outcome prediction and severity adjustment that informs trauma quality assessment and research. Discrimination and precision of such systems is tested in validation studies. The German TraumaRegister DGU^®^ (TR-DGU) and the Trauma Audit and Research Network (TARN) from the UK agreed on a cross-validation study to validate their prediction scores (RISC II and PS14, respectively).

**Methods:**

Severe trauma patients with an Injury Severity Score (ISS) ≥ 9 documented in 2015 and 2016 were selected in both registries (primary admissions only). The predictive scores from each registry were applied to the selected data sets. Observed and predicted mortality were compared to assess precision; area under the receiver operating characteristic curve was used for discrimination. Hosmer–Lemeshow statistic was calculated for calibration. A subgroup analysis including patients treated in intensive care unit (ICU) was also carried out.

**Results:**

From TR-DGU, 40,638 patients were included (mortality 11.7%). The RISC II predicted mortality was 11.2%, while PS14 predicted 16.9% mortality. From TARN, 64,622 patients were included (mortality 9.7%). PS14 predicted 10.6% mortality, while RISC II predicted 17.7%. Despite the identical cutoff of ISS ≥ 9, patient groups from both registries showed considerable difference in need for intensive care (88% versus 18%). Subgroup analysis of patients treated on ICU showed nearly identical values for observed and predicted mortality using RISC II.

**Discussion:**

Each score performed well within its respective registry, but when applied to the other registry a decrease in performance was observed. Part of this loss of performance could be explained by different development data sets: the RISC II is mainly based on patients treated in an ICU, while the PS14 includes cases mainly cared for outside ICU with more moderate injury severity. This is according to the respective inclusion criteria of the two registries.

**Conclusion:**

External validations of prediction models between registries are needed, but may show that prediction models are not fully transferable to other health-care settings.

## Introduction

Severe trauma is a major health problem where a lot of younger people are affected, as many would die or suffer from long-term disability. Therefore, improvement of treatment and outcome is an important objective. Trauma registries are established in hospitals, regions or countries. They document patient’s demographics, circumstances of the accident, injuries, physiological reactions, pre-hospital and in-hospital care and outcome. This allows quantification of the burden of injuries, and to compare it with the observed outcome. Based on these comparisons, treatment options or diagnostic modalities may be evaluated. The performance of hospitals can also be evaluated when observed mortality is compared to the expected outcome.

The TraumaRegister DGU^®^ (TR-DGU) of the German Trauma Society (DGU, Deutsche Gesellschaft für Unfallchirurgie) and the Trauma Audit and Research Network (TARN) in the UK are trauma registries that have existed for more than 25 years. They provide regular quality audits and benchmarking data for their participating hospitals.

The main outcome measure for severely injured patients is still mortality. Mortality rates, however, depend on multiple factors, including the patient’s condition (age, sex, existing diseases), the type of trauma (blunt or penetrating), the injuries (severity, number, and pattern), and the patients’ physiological reactions to these injuries (shock, unconsciousness, coagulopathy, etc.). Case-mix adjustment enables comparison of hospital’s performance on a ‘like with like’ basis. Unadjusted mortality rates, for example, would penalize the work of large trauma centers where the most severe cases are treated. Susan Baker, who first developed the Injury Severity Score (ISS), once quoted: ‘If you have never felt the need for any type of severity scoring system, then you probably have never had to explain how it is that the survival rate of 85% in your trauma center is actually better than the survival rate of 97% in some other hospital where the patients are much less seriously injured” [1]

Prognostic scoring systems using case-mix adjustment provide an estimate of the risk of death for each individual patient. Patient groups could then be evaluated by comparing their observed mortality rate with their average prognosis.

Both large trauma registries described above use such tools for outcome adjustment. The TR-DGU initially used the TRISS score for outcome adjustment [2]. In 2003, it changed to the Revised Injury Severity Classification (RISC) score which has been developed and validated with data from the TR-DGU [3]. In 2013, an update of this score has been developed based on data from 2010–11, with validation on 2012 data [4]. The revised RISC II score has been used in the annual reports of the registry since 2013. TARN has also developed a prognostic score, which has evolved from the TRISS and has been used for quality assurance trauma care in the UK since 2004. The coefficients of this score are routinely updated to increase the precision of the prediction. The most recent version of this score is from 2017, but this paper uses the PS14 coefficients from the original publication [5]. The risk prediction models are described in the appendices. Table 1 highlights the fact that the inclusion criteria for both registries differ somewhat as do the variables utilized in risk adjustment. The TARN model has been validated in the National Trauma Databank [6], and the RISC II score has been validated repeatedly in TR-DGU data [3] and in Spanish [7] and Finish [8] data. However neither model has been previously tested in an external large European Trauma Registry, but this is valuable to understand their external validity.

**Table 1:** Patient inclusion criteria of both registries, and list of variables needed for score calculation (for details of score calculation, see “Appendix 1” and “Appendix 2”)

	TraumaRegister DGU (TR-DGU)	Trauma Audit and Research Network (TARN)
Founded	1993	1989
Inclusion criteria	Alive on admission Trauma team activation Admission via shock room Need for intensive care (or death before ICU admission)	At least one of the following Hospital admission ≥ 3 days Intensive care admission Transfer to a tertiary/specialist center In-hospital death within 30 days
Exclusions	Pre-hospital deaths Severe burns Drowning, poisoning, hanging Isolated femur fractures	Pre-hospital deaths Isolated femoral neck or single pubic ramus fracture in patients > 65 years Simple isolated injuries
Outcome prediction model	Revised Injury Severity Classification, version II (RISC II)	Probability of Survival model, version 2014 (PS14)
Predictors	Injury severity (worst AIS; second worst AIS; head AIS) Age Gender Pupil size and reactivity [11] Penetrating mechanism Pre-injury ASA GCS motor function Cardio-pulmonary resuscitation Blood pressure Base deficit Hemoglobin Int. Normalized Ratio (INR)	Injury severity (ISS) Age Gender Glasgow Coma Scale (GCS) Charlson Comorbidity Index (CCI)

The aim of this paper is to perform a cross-validation of both prognostic scoring systems using data from both registries, where score-based prognoses will be compared with the observed outcome.

## Methods

### Registries

The TraumaRegister DGU^®^ of the German Trauma Society (Deutsche Gesellschaft für Unfallchirurgie, DGU) was founded in 1993. The aim of this multi-center database is a pseudonymized and standardized documentation of severely injured patients. Data are collected prospectively in four consecutive time phases from the site of the accident until discharge from hospital: (A) pre-hospital phase, (B) emergency room and initial surgery, (C) intensive care unit (ICU) and (D) discharge. The documentation includes detailed information on demographics, injury pattern, comorbidities, pre- and in-hospital management, course on intensive care unit, relevant laboratory findings including data on transfusion and outcome of each individual. The inclusion criterion is admission to hospital via the emergency room with subsequent admission to intensive care. Patients who reached the hospital alive but died before ICU admission are included as well (Table 1). Injuries are coded according to a reduced version of the Abbreviated Injury Scale (AIS) version 2005/update 2008, where injuries of the same severity level were merged. The infrastructure for documentation, data management, and data analysis is provided by AUC—Academy for Trauma Surgery (AUC—Akademie der Unfallchirurgie GmbH), a company affiliated to the German Trauma Society. The scientific leadership is provided by the Committee on Emergency Medicine, Intensive Care and Trauma Management (Sektion NIS) of the German Trauma Society. The participating hospitals submit pseudonymized data into a central database via a web-based application. Scientific data analysis is approved according to a peer review process established by Sektion NIS. The participating hospitals are primarily located in Germany (90%), but a rising number of hospitals of other countries contribute data as well (at the moment from Austria, Belgium, China, Finland, Luxembourg, Slovenia, Switzerland, The Netherlands, and the United Arab Emirates). Currently, approximately 35,000 cases from more than 600 hospitals are entered into the database per year. Participation in TraumaRegister DGU^®^ is voluntary. For hospitals associated with TraumaNetzwerk DGU^®^ however, the entry of at least a basic data set is obligatory for reasons of quality assurance.

TARN is an independent trauma audit which was founded in 1989. TARN is part of the University of Manchester. TARN is funded by its member National Health Service (NHS) and European hospitals by annual subscriptions, where the fees are based on their annual accident and emergency (A&E) attendance. TARN is overseen by a board consisting of health professionals from different backgrounds, specialties, skills and geography. The TARN Board meets twice a year to approves the 5-year strategic plan for TARN, agrees annual budget and fee structure and evaluates the performance of the research group and the performance of the Clinical Audit Group. Data are recorded online through the bespoke TARN website. Every injury is recorded and defined according to the Abbreviated Injury Scale (AIS) dictionary version 2005/update 2008. This is used by trained coders to enable calculation of the Injury Severity Score (ISS). All trauma receiving hospitals in England, Wales and Northern Ireland are members, and also foreign hospitals from the Republic of Ireland, Copenhagen and Bern. There are approximately 230 hospitals contributing with 85,000 submissions annually. The inclusion criteria of TARN database are listed in Table 1.

### Patients

Patients from both registries with an Injury Severity Score (ISS) ≥ 9 points were included and restricted to patients treated in German or English hospitals, respectively. Patients transferred in from other hospitals were excluded, since the initial treatment may have altered their physiology. Patients who were transferred out within 48 h were also excluded, since outcome was considered unknown. Finally, the age, the injury codes, and the survival status have to be available. Table 2 shows the selection of cases from both registries. All data were selected from a 2-year period (January 2015–December 2016).

**Table 2:** Patient selection flowchart

	TR-DGU	TARN
Patients documented in 2015–2016	81,479^b^	111,265^b^
Exclusions^a^		
Non-German/non-UK	− 10,917	− 8344
ISS < 9	− 23,660	− 25,101
Transfer in cases	− 6289	− 11,948
Early transfer out cases	− 5353	− 1250
Missing age, ISS, or outcome	45	0
Study population	40,638	64,622
Patients with intensive care treatment	35,803	11,744

^a^Multiple reasons for exclusions may apply

^b^After excluding isolated hip, burns, hanging and drowning

A subgroup analysis based on patients treated in the intensive care unit (ICU) was carried out, as this was where a relevant difference between both data sets existed. This subgroup excluded TARN cases where no intensive care stay was observed, and patients from both registries who died before ICU admission.

### Statistics

In both registries, the predicted mortality rate from RISCII and PS14 were calculated according to the published rules (see “Appendix 1” and “Appendix 2”). As some of the predictors were different, a mapping exercise was carried out. For example, pre-existing diseases were coded as pre-trauma ASA level in TR-DGU which was recommended in the Utstein European Core Dataset [9]. TARN used the Charlson Comorbiditiy Index (CCI) for grading the severity of pre-existing diseases [10]. This adjustment was based on the prevalence and mortality rates of patients with specific CCI or ASA levels. For example, CCI 0 was considered equivalent to ASA 1; CCI 1–5 to ASA 2; etc.

In both registries, the 30-day mortality was chosen as the primary outcome measure. Patients who were discharged alive earlier than 30 days after admission were considered as survivors. Patients who died in hospital beyond day 30 were considered as survivors in this analysis. However, both hospital and 30-day mortality were reported.

The predicted mortality rate was calculated as the mean value across all patients and was presented as a percentage. The precision of each predictive score (in each database) is assessed from the comparison with the actually observed mortality rate. The discrimination of a score describes the ability to give different predictions for survivors and non-survivors. The area under the receiver operation characteristics (ROC) curve is considered a summary measure for discrimination. For this value a 95% confidence interval was determined. But outcome prediction should not only be precise on average, i.e., in the whole group of patients, but also in subgroups with high and low risk of death. Calibration is usually measured with Hosmer–Lemeshow (H–L) goodness‐of‐fit statistics. It summarizes deviations from observed versus expected mortality rates in ten subgroups of increasing risk of death. A score should thus not only have a good performance on average, but also for high- and low-risk patients. The lower the H–L statistic, the better the calibration. Due to the huge number of cases in both registries. The respective *p* value should not be over-interpreted.

Analysis was performed using SPSS statistical software (version 24, IBM Inc., Armonk, NY, USA, for TR-DGU data) and Stata 14 (StataCorp. 2015. Stata Statistical Software: Release 14. College Station, TX: StataCorp LP for TARN data).

## Results

During the 2 years observation period, 64,622 trauma patients from TARN and 40,638 patients from TR-DGU were available for this analysis. An overview about the basic characteristics of both data sets is given in Table 3. Patients in the TR-DGU were on average 10–15 years younger and 16% more likely to be male than in TARN (Table 1). Road traffic collision was the most frequent mechanism in TR-DGU, while most patients in TARN were low falls. The rates of head injuries were comparable, but TR-DGU patients suffered much more frequently from thoracic trauma (45% versus 27%). Patients from TR-DGU had a higher injury severity (19 versus 15 points) and 30-day mortality rate was 2.3 percentage points higher in TR-DGU. Late deaths beyond day 30 in hospital were a rare event in both registries (0.5% in TR-DGU and 1.0% in TARN). Eighty-eight percent of TR-DGU patients were treated on ICU. While only 18% of TARN patients required intensive care.

**Table 3:** Basic characteristics of included trauma patients

Registry	All patients		ICU patients only	
	TR-DGU	TARN	TR-DGU	TARN
Number of cases	40,638	64,622	35,803	11,744
Age (years)^a^	52.6/54 (22.3)	62.1/68.6 (25.3)	52.5/54 (22.3)	51.4/50.7 (23.4)
Male patients	69.6%	53.2%	70.2%	71.4%
Pre-existing diseases^b^	18.4%	14.7%	18.8%	10.4%
Penetrating trauma	4.0%	2.7%	3.9%	7.3%
Road traffic accident	53.3%	21.1%	53.5%	39.3%
Low fall^c^	24.1%	57.9%	24.0%	28.2%
Transportation by helicopter	21.4%	6.4%	22.8%	20.0%
Injury Severity Score (ISS)^a^	19.9/17 (11.3)	15.7/13 (8.8)	20.2/17 (10.9)	21.0/22.6 (11.9)
Relevant head injury (AIS ≥ 3)	37.7%	34.3%	40.1%	43.1%
Relevant thoracic injury (AIS ≥ 3)	44.9%	27.2%	45.5%	50.7%
Relevant abdominal injury (AIS ≥ 3)	10.5%	4.4%	10.8%	14.4%
Relevant extremity injury (AIS ≥ 3)	28.1%	32.3%	27.0%	19.5%
Isolated head injury (AIS head ≥ 3, else ≤ 1)	14.3%	25.3%	15.1%	21.7%
Treated on ICU	88.1%	18.2%	100%	100%
Length of stay in hospital (days)^a^	16.5/12 (16.5)	16.5/10 (21.8)	17.5/13 (17.0)	13/22.9 (31.3)
Died within 24 h	5.8%	2.2%	4.3%	3.2%
30 days mortality^d^	11.7%	9.7%	10.8%	17.2%
Hospital mortality	12.2%	10.7%	11.4%	18.5%

^a^Continuous measures were presented as mean/median (standard deviation)

^b^TR-DGU: pre-injury ASA 3–4; TARN: Charlson Comorbidity Index 6 +

^c^TR-DGU: < 3 m; TARN: < 2 m

^d^Patients discharged after day 30 were considered as survivors

### Results from TR-DGU

**Fig. 1:**
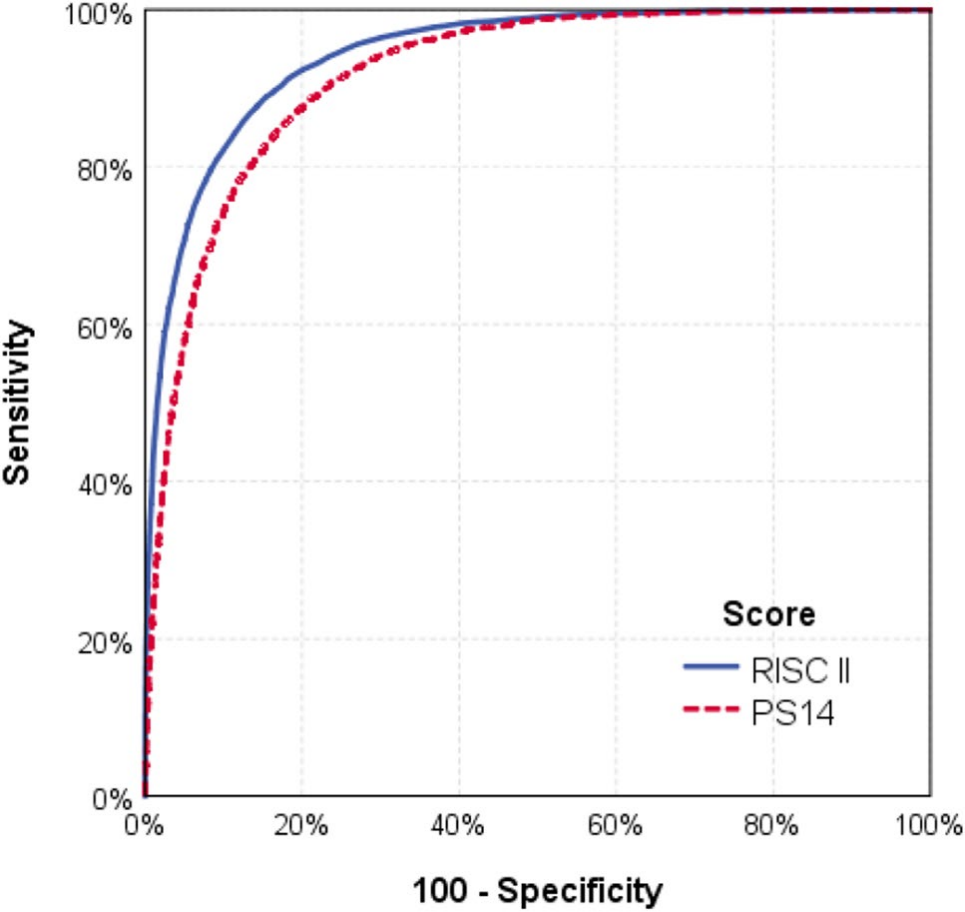
ROC curves for RISC II and PS14 using all patients from the TR-DGU data set (*n* = 40,638). The areas under the curves were 0.933 and 0.918, respectively

**Fig. 2:**
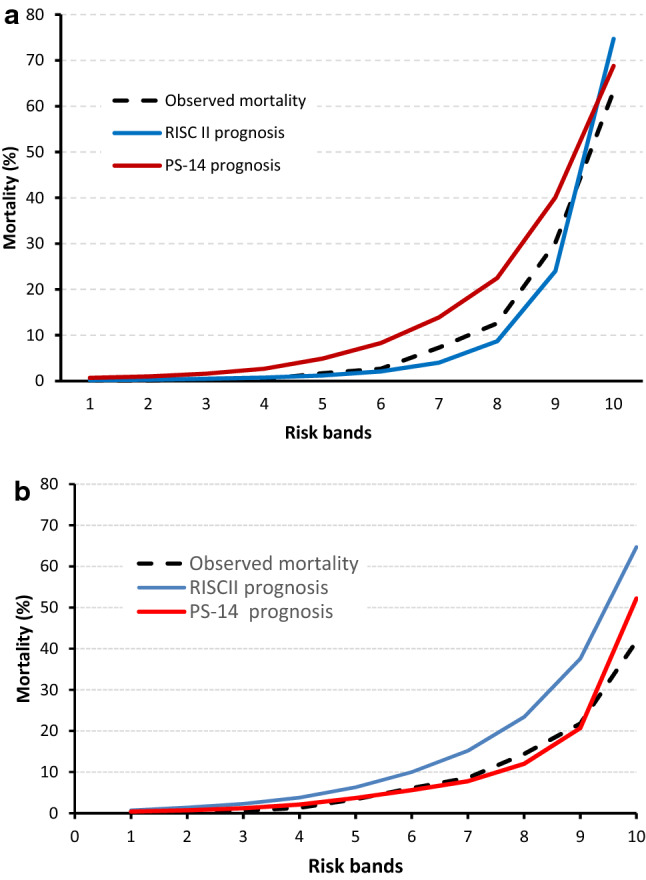
**a** Observed and predicted mortality in ten subgroups of equal size (risk bands according to PS14, *n* = 39,295) in TR-DGU. **b** Observed and predicted mortality in ten subgroups of equal size (risk bands according to RISCII, *n* = 64,622) in TARN

The RISC II score could be calculated in all patients, since only injury pattern and age were required as mandatory variables. However, on average 11.8 of 13 variables per patient used in the RISC II were available in TR-DGU. No imputation was performed; each score component has a category for missing information (see “Appendix 1”). Predicted mortality (11.2%) matched well with the observed mortality (11.7%), while PS14 predicted 16.4% deaths (Table 4). A few cases were excluded from PS14 calculation due to missing Glasgow Coma Scale data (no imputation of missing values performed). The area under the ROC curve was higher for RISC II (0.933) than for PS14 (0.918, Fig. 1), and also H–L statistic was much lower for RISC II in the German data (Fig. 2a).

**Table 4:** Observed (30 days) and predicted mortality based on RISC II and PS14 in all patients and the subgroup with intensive care

Registry	All patients		ICU patients only	
	TR-DGU	TARN	TR-DGU	TARN
RISC II				
Number of cases with prognosis	40,638 (100%)	64,622 (100%)	35,803 (100%)	11,744 (100%)
Observed mortality (30 days)	11.7%	9.7%	10.8%	17.5%
Expected mortality	11.2%	17.7%	11.2%	17.2%
Expected mortality in survivors	6.2%	14.5%	6.2%	12.2%
Expected mortality in non-survivors	52.1%	47.0%	52.1%	43.4%
Area under the ROC curve	0.933	0.861	0.933	0.867
95% confidence interval	0.929–0.937	0.857–0.866	0.929–0.937	0.858–0.875
H–L goodness-of-fit statistic	92.1; *p* < 0.001	4405.1; *p* < 0.001	81.7; *p* < 0.001	25.5; *p* = 0.005
PS14				
Number of cases with prognosis	39,489 (97.2%)	61,950 (95.9%)	34,881 (97.4%)	11,529 (98.2%)
Observed mortality (30 days)	12.4%	9.7%	11.5%	17.2%
Expected mortality	16.9%	10.6%	17.2%	16.2%
Expected mortality in survivors	11.1%	7.6%	11.9%	9.3%
Expected mortality in non-survivors	58.3%	38.5%	57.4%	42.8%
Area under the ROC curve	0.918	0.885	0.908	0.863
95% confidence interval	0.914–0.921	0.881–0.889	0.904–0.912	0.855–0.872
H–L goodness-of-fit statistic	1215.7; *p* < 0.001	126.8; *p* < 0.001	1640.4; *p* < 0.001	95.7; *p* < 0.001

### Results from TARN

In the TARN data the availability of prognoses was similar to TR-DGU for both scores. Outcome prediction with PS14 (10.6%) was close to the observed mortality rate of 9.7%. The RISC II score predicted nearly twice as many deaths (17.7%) than observed (9.7%). The areas under the ROC curves was higher for PS14 (0.885) when compared with RISC II (0.861). The H–L statistic for RISC II was much higher than that of PS14 (3382 vs*.* 129, Table 4 and Fig. 2b). Due to the large sample size, all H–L statistics were significant.

### Subgroup with intensive care

Subgroup analysis in patients who needed intensive care left 35,803 patients from TR-DGU and 11,744 patients from TARN. The demographic and injury characteristics of these groups were similar. Mean age differed by only 1 year, and ISS by 0.8 points (Table 3). The difference in prevalence of head trauma was only 3%, in thoracic trauma 5%, and 4% in abdominal trauma. The observed 30 days mortality rate was 10.8% in TR-DGU and 17.2% in TARN. The expected mortality based on PS14 was very similar in both registries: 17.2% in TR-DGU patients, and 16.2% in TARN patients (Table 4). The RISC II score predicted a mortality of 11.2% and 17.2%, respectively. This was within 1% deviation range from the observed mortality in both registries.

## Discussion

Trauma scoring systems combine findings known to be associated with a good or bad outcome into a single value which could be transformed into a risk of death estimator. Such findings could be based on the patient (age, pre-existing diseases), the injury mechanism (blunt/penetrating), type and severity of injuries (ISS, head injury), and the actual physiology (shock, unconsciousness, coagulopathy). They are usually developed using multivariate models to find the appropriate weights for each of their components. Obviously, such models will provide an optimal adaptation to the development data set. Therefore, independent validation studies are necessary.

Both scores considered in this study have been developed and validated within their own registries. Such a validation has also been repeated here: the RISC II score has been applied to TR-DGU patients from 2015 to 16, and the PS14 has been applied to new TARN patients as well. As expected, the performance of both scores is quite good: predicted and observed mortality lie within a range of 1%. This could mostly be explained by the fact that the system (definition of variables; inclusion of patients; mode of data collection) has not changed. But what happens if a score is applied in a different setting?

The RISC II has been applied to British TARN patients, and the PS14 has been applied to German TR-DGU patients. In both settings, the performance was poor. The prediction of PS14 (16.9% mortality) was higher than observed (12.4%) in German patients, and the performance of RISC II was even worse in English patients (predicted 17.7% versus observed 9.7%).

One reason for this mismatch may be the large differences in the patient groups, despite the uniform requirement of ISS ≥ 9. A striking difference was the need for intensive care, which is a pragmatic inclusion criterion for TR-DGU. Although the criteria for intensive care vary from country to country, only 18% of the TARN patients received intensive care. This small subgroup was quite comparable to the German patients in terms of age, ISS, and injury pattern. In that subgroup, the RISC II showed much better results (observed and predicted mortality differed by 0.3 percentage points only). This may also explain the bad performance in all TARN patients. Many of the TARN patients not admitted to ICU had missing laboratory values (completeness of base excess, INR, hemoglobin was < 5%). The RISC II assumes some average values there, but these average values were based on severe cases from the development data set. ICU admission is an inclusion criterion for TR-DGU. Thus many TARN patients with missing laboratory values (not treated on ICU) received a worse prognosis. If these laboratory values would have been available (and in a normal range), then risk of death based on RISC II would decrease. Thus, the application of RISC II in less severe cases with several missing data is not recommended and may lead to false high risk of death estimates*.* For application in non-ICU patients, the RISC II score seems inappropriate, or needs adaptation.

While RISC II performed well in British ICU patients, PS14 did not in German patients. The main reason for PS14 to not perform well in the TR-DGU is the inclusion criteria. The PS model was developed on a general trauma population which included patients with low severity, patients who were transferred from other hospitals after being stabilized and would have a higher chance of survival. The mortality rate in the TARN general population is around 6%; that is why it probably overestimates mortality in the TR-DGU setting.

There were also some differences in data collection. Pre-existing diseases, for example, are recorded with the CCI in TARN, while TR-DGU uses the pre-trauma ASA. Laboratory values are also rarely documented in TARN; such values may improve the prediction but cause a problem when missing. Furthermore, injury coding in TARN is done by experienced coders, while TR-DGU applies an online coding tool with a simplified AIS system. The PS14 score requires five variables only (ISS, GCS, CCI, age and gender), while the RISC II combines 13 variables. The PS14 has been developed using all TARN patients, including transfers, and not just the selected cases here (primary admission with ISS ≥ 9, or intensive care). Thus performance of PS14 is expected to be better in a large data set that also includes moderate trauma. The RISC II, on the other hand, considered patients with ISS ≥ 4 in its development set, but only those with need of intensive care. All these differences might affect the prognoses.

Finally, there might also be differences in outcome in the two countries. Both are developed countries, but the health system is different. This refers to the pre-hospital care (doctor versus paramedic at scene); emergency departments; the role of orthopedic or trauma surgery; intensive care; and the financing system.

Although risk prediction scores should aim to improve their precision, and cross-validation studies like the present one will contribute to this, the most important use of these tools would be an ongoing comparison over time, within one organization or hospital, with the aim to improve the observed/expected ratio and thus the outcome of severely injured patients

External validations of prediction models between registries like this one are needed, but may show that prediction models are not fully transferable to other health-care settings.

## Appendix 1: RISC II prognostic model of TR-DGU

**Table:**

Variable	Description
Worst and second worst injury	AIS injury severity level; if only one injury was coded, the second worst injury was set to zero
Head injury	AIS injury severity level of the body region ‘head’ as defined for ISS
Age	Age in years at the time of accident, 10 categories
Sex	Male/female
ASA	Pre-trauma ASA (American Society of Anaesthesiologists) score, as defined in the Utstein European core data set [9]
Pupil reactivity and size	Three categories according to the Eppendorf Cologne Scale [11]. The first pre-hospital assessment was used; if missing, assessment on admission was used
Motor function	Motor function from Glasgow Coma Scale, but reduced to 4 categories: normal; directed; non-directed; and none. The first pre-hospital assessment, if missing assessment on admission in non-intubated cases
Mechanism	Blunt/penetrating
Blood pressure	Systolic blood pressure (mmHg), first measurement after admission; in case of missing values, the first pre-hospital measurement
Coagulation: INR	International Normalized Ratio (INR); first measurement after admission
Base deficit	Base deficit, or base excess (mEq/l); first measurement after admission
Hemoglobin	Hemoglobin (g/dl); first measurement after admission
CPR	Pre-hospital cardio-pulmonary resuscitation (CPR) in case of cardiac arrest

The ‘???’ below indicates the category used for missing values (coefficient is always 0).

## Appendix 2: PS14 predictive model of TARN

**Table:**

Predictors	Coefficients	*p* value	95% CI for odds ratios	
$\sqrt {\frac{10}{{{\text{ISS}}}}} - 0.8686$	− 2.79052	< 0.001	0.0464	0.0812
$\log_{e} \left( {\frac{{\text{ISS}}}{{10}}} \right) - 0.2817$	− 2.57574	< 0.001	0.0659	0.0879
GCS = 3	− 3.79637	< 0.001	0.0203	0.0248
GCS 4–5	− 2.73865	< 0.001	0.0557	0.0751
GCS 6–8	− 1.87664	< 0.001	0.1361	0.1722
GCS 9–12	− 1.29443	< 0.001	0.2477	0.3033
GCS 13–14	− 0.46062	< 0.001	0.5853	0.6801
GCS 15 (reference)	0.00000			
GCS “Intubated”	− 2.62397	< 0.001	0.0595	0.0884
CCI notknown	− 0.44900	< 0.001	0.5919	0.6882
CCI 0 (reference)	0.00000			
CCI 1–5	− 0.49572	< 0.001	0.5692	0.6519
CCI 6–10	− 0.96308	< 0.001	0.3474	0.4195
CCI > 10	− 1.59703	< 0.001	0.1791	0.2289
Age 0–5	− 0.00483	0.9770	0.7206	1.3745
Age 6–10	0.25323	0.2750	0.8174	2.0300
Age 11–15	− 0.08435	0.5780	0.6825	1.2378
Age 16–44 (reference)	0.00000			
Age 45–54	− 0.41388	< 0.001	0.5795	0.7542
Age 55–64	− 0.93229	< 0.001	0.3457	0.4482
Age 65–74	− 1.58082	< 0.001	0.1814	0.2335
Age ≥ 75	− 2.67520	< 0.001	0.0621	0.0765
Gender MALE (reference)	0.00000			
Gender female	− 0.17252	0.0290	0.7211	0.9821
Age 0–5 × female	− 0.13805	0.5820	0.5322	1.4255
Age 6–10 × female	0.43973	0.3200	0.6518	3.6970
Age 11–15 × female	0.21675	0.4630	0.6961	2.2160
Age 45–54 × female	− 0.06972	0.6000	0.7183	1.2110
Age 55–64 × female	0.17164	0.1590	0.9350	1.5075
Age 65–74 × female	0.25829	0.0220	1.0376	1.6155
Age ≥ 75 + × female	0.34770	< 0.001	1.1928	1.6806
Constant	5.28621	< 0.001		
